# A Metal–Dielectric Photonic Crystal and Its Phase Response Utilized in an Ultra-High-Performance Humidity Sensor

**DOI:** 10.3390/s26196255

**Published:** 2026-10-02

**Authors:** Jakub Chylek, Michal Gryga, Dalibor Ciprian, Petr Hlubina

**Affiliations:** Department of Physics, Technical University of Ostrava, 17. Listopadu 2172/15, 708 00 Ostrava-Poruba, Czech Republic; michal.gryga@vsb.cz (M.G.); dalibor.ciprian@vsb.cz (D.C.)

**Keywords:** metal–dielectric photonic crystal, Bloch-like surface waves, phase interrogation, refractive index, relative humidity, sensitivity, figure of merit, limit of detection

## Abstract

Metal–dielectric photonic crystals (MDPhCs) generating Bloch-like surface waves (BLSWs) are accompanied by narrow resonance dips and sensitivities to gaseous analytes outperforming the BSW-based sensors. In this theoretical paper, we report on a sensing concept based on the phase interrogation and BLSWs supported by an MDPhC in the Kretschmann configuration when the sensing characteristics are substantially improved. For an MDPhC comprising two bilayers of TiO_2_/Ag with a termination layer of TiO_2_, we show that the BLSW-based phase response is characterized by a steep and abrupt phase difference between the TM and TE waves. It was revealed that for the refractive index (RI) change in a range of 1.0002727–1.0003751 RI units (RIU) or, equivalently, for a relative humidity (RH) change in air in a range of 0–100 %RH, the sensitivities to the RI and RH are up to 70 μm/RIU and 0.072 nm/%RH, respectively, and the figures of merit (FOMs) are up to 700,000 RIU^−1^ and 0.717 %RH^−1^, respectively. In addition, the limits of detection (LODs) reach 1.4 × 10^−7^ RIU and 0.14 %RH, respectively. We also evaluated the phase difference at specific wavelengths, and the phase sensitivities to the RI and RH at a wavelength of 1637.92 nm are as high as 4.7 × 10^7^ deg/RIU and 47.7 deg/%RH, respectively. The corresponding LODs are as low as 2.1 × 10^−10^ RIU and 2.1 × 10^−4^ %RH, respectively. This research demonstrates that the BLSW-based sensors employing MDPhCs and utilizing the phase interrogation exhibit outstanding sensing characteristics, and they can be used in a simple, ultra-high-performance humidity sensing.

## 1. Introduction

The relative humidity (RH) of air is a physical quantity which is important in many fields, including meteorological services, agricultural production, food manufacturing and storage, pharmaceutical manufacturing, and health monitoring. Various types of humidity sensors have been proposed and realized, such as electrical, triboelectrical, electrochemical, frequency, and optical ones [1,2,3,4,5,6,7,8]. For these sensors, the specifications of parameters and characteristics, such as the response time, measurement range, hysteresis, linearity, and sensitivity, are important. To the simplest RH sensors belong electrical sensors utilizing changes in electric capacity or resistance in a sensing element with the RH of the air. Unfortunately, the electrical RH sensors usually show hysteresis when subjected to a high-humidity environment for a long time. However, the response of such sensors could be affected by electromagnetic interferences, and in this regard, optical humidity sensors [2,9,10,11,12,13,14], especially fiber optic sensors [10,11,12,15] are preferred due to their advantages such as remote measurement, high sensitivity, and electromagnetic immunity.

Highly promising optical humidity sensors are those based on one-dimensional photonic crystals (1DPhCs) that are mechanically and chemically robust and can also be applied in aggressive environments [16]. Truncated 1DPhCs [17], as complex dielectric structures [18] with periodically varying dielectric constants, are characterized by a photonic band gap (PBG). If a 1DPhC is composed of bilayers of dielectric media of high and low refractive indices (RIs) and a termination layer as well, then the optical waves in the 1DPhC are gradually enhanced with the maximum amplitude near the interface with the outer medium (analyte). These waves are referred to as Bloch surface waves (BSWs), and they enable high optical field confinement and are accompanied by narrow resonance dips within the PBG of the reflection spectrum. Narrow BSW-based resonances are advantageous compared with wide resonances accompanied by the surface plasmon resonance (SPR) phenomenon [19,20,21,22,23], and they are preferred in the realization of various types of optical sensors [24,25,26,27,28,29], including gas sensors [30,31,32] with a porous termination layer (PTL) [33,34]. Contrary to surface plasmon waves, BSWs can be excited by both TE and TM waves [35,36] in various spectra regions, including near- or mid-infrared regions [37,38].

From the experimental point of view, free space excited sensors are preferable, but they are not possible for BSW-based sensors. Fortunately, alternative sensors based on confined electromagnetic modes called Tamm plasmons (TPs) [39,40,41] are possible. A Tamm plasmon is generated when a 1DPhC incorporates a thin metal layer, and similar to the BSW, the optical wave is gradually enhanced in the 1DPhC, but its maximum amplitude is near the interface of the 1DPhC and metal layer. TPs can be excited in the spectral domain at any angle of incidence by both TE and TM waves, and this advantage has led to applications in the field of gas sensors [34,42,43,44,45,46,47]. However, the fact that the confined optical field is located within the structure near the metal–dielectric interface limits the TP-based sensors. To overcome this fact, porous silicon (PSi) in a 1DPhC [47] is employed, and the analyte can reach the detection volume [34,42,47,48,49,50]. In addition, TP- or BSW-based gas sensors can be operated using not only the angular [32] or wavelength interrogations [34,47,48,49,50] but also the phase interrogation [33,51,52].

It is well known that the limit of detection (LOD) of phase-based measurements [53,54,55] outperforms amplitude-based ones by at least two orders of magnitude. This is due to a maximal phase change that occurs at the very center of the resonance dip [56] and is conditioned by almost zero reflection [57,58,59,60]. As an example, a simple metal–dielectric multilayer thin-film stack has been analyzed [58], and the structure showed an abrupt phase change at the reflectionless point due to the presence of a highly absorbing ultra-thin film of germanium in the stack. Moreover, the concept of phase-sensitive detection has received much attention for relative humidity (RH) sensing [52]. Narrow resonance curves also mean that the figure of merit (FOM) is significantly improved, as demonstrated for the BSW-based optical sensors that can utilize not only the wavelength interrogation, based on variation in the resonance dip with the analyte RI change, but also the phase interrogation [36,61,62,63,64,65,66,67,68,69,70], based on an abrupt spectral phase change within the resonance curve.

Metal–dielectric photonic crystals (MDPhCs) [71,72,73] generating Bloch-like surface waves (BLSWs) [74,75] have emerged as an alternative to 1DPhCs supporting BSWs at the interface with the analyte. The BLSWs are accompanied by narrow resonance dips and sensitivities to the RI of gaseous analytes outperforming the BSW-based sensors. It was revealed that the sensitivity and FOM are up to 28 μm/RI unit (RIU) and 1217 RIU^−1^, respectively, and the LOD is as low as 3.6 × 10^−6^ RIU [74]. If we concentrate on the phase response and thus on the phase interrogation, the sensing characteristics can be substantially improved in comparison with the reflectance response utilized in the wavelength interrogation.

In this theoretical paper, an MDPhC generating the BLSW in the Kretschmann configuration is employed in humidity sensing when the sensing concept based on the phase interrogation is utilized, and the sensitivity, FOM, and LOD are substantially improved. We show that the BLSW-based phase response for an MDPhC, comprising bilayers of TiO_2_/Ag with a termination layer of TiO_2_ is characterized by the sensitivities to the RI and RH of air up to 70 μm/RIU and 0.072 nm/%RH, respectively. In addition, the FOMs are up to 700,000 RIU^−1^ and 0.717 %RH^−1^, respectively, and LODs are as low as 1.4 × 10^−7^ RIU and 0.14 %RH, respectively. Extending the analysis to evaluate the phase difference at a specific wavelength, the phase sensitivities to the RI and RH of air are enhanced, and the LOD is lowered as well but within a narrow RI range. This sensing concept is demonstrative for optical humidity sensors and has the potential to be applied in gaseous analyte sensing.

## 2. Theoretical Model

Light propagation through an MDPhC in the Kretschmann configuration can be analyzed by the transfer matrix method (TMM) [76]. The method enables expressing not only the spectral reflectances $R_{s,p}\left(\lambda \right)$ for both *s*-polarized (transverse electric (TE)) and *p*-polarized (transverse magnetic (TM)) waves but also their spectral phase difference, known as the ellipsometric phase $\Delta \left(\lambda \right)=\phi_{p}\left(\lambda \right)-\phi_{s}\left(\lambda \right)$, where $\phi_{p}\left(\lambda \right)$ and $\phi_{s}\left(\lambda \right)$ are phases of *p*- and *s*-polarized waves, respectively. If *N* layers are considered, including both metal and dielectric media, the propagation matrices in different layers and the transmission matrices across different interfaces can be used to express the total transfer matrix M(λ) at the wavelength λ as follows:

(1) $$M\left(\lambda \right)=\left[\begin{array}{cc} M_{11}\left(\lambda \right) & M_{12}\left(\lambda \right)\\ M_{21}\left(\lambda \right) & M_{22}\left(\lambda \right) \end{array}\right],$$

and it can be expressed by means of both the boundary and propagation matrices for *s*- and *p*-polarized waves, as specified in [74].

The total transfer matrix elements enable expressing the complex reflection coefficient r(λ) as follows:

(2) $$\begin{array}{c} r\left(\lambda \right)=\frac{M_{21}\left(\lambda \right)}{M_{11}\left(\lambda \right)}, \end{array}$$

and it can be specified for both *s* and *p* polarizations as follows:

(3) $$r_{s,p}\left(\lambda \right)=\left|r_{s,p}\left(\lambda \right)\right|e^{i\phi_{s,p}\left(\lambda \right)},$$

with the spectral reflectances $R_{s,p}\left(\lambda \right)$ being

(4) $$R_{s,p}\left(\lambda \right)={\left|r_{s,p}\left(\lambda \right)\right|}^{2},$$

and with the spectral phase difference Δ(λ) being

(5) $$\Delta \left(\lambda \right)=\phi_{p}\left(\lambda \right)-\phi_{s}\left(\lambda \right).$$

### Materials

The MDPhC under study, whose optical response to humid air is analyzed in the Kretschmann configuration by employing the phase interrogation, is shown schematically in the next section. It consists of a glass substrate, with titanium dioxide and silver layers.

We consider that both a prism and substrate are made from BK7 glass, with its RI as a function of the wavelength described by a three-term Sellmeier formula [77]:

(6) $$n_{\mathrm{BK}7}^{2}\left(\lambda \right)=1+\sum_{i=1}^{3}\frac{A_{i}\lambda^{2}}{\lambda^{2}-B_{i}},$$

where the wavelength λ is in μm and the values of the Sellmeier coefficients at room temperature are as follows: $A_{1}$ = 1.03961212, $A_{2}$ = 2.31792344 $\times \;10^{-1}$, $A_{3}$ = 1.01046945, $B_{1}$ = 6.00069867 $\times \;10^{-3}\;\mu$m^2^, $B_{2}$ = 2.00179144 $\times \;10^{-2}\;\mu$m^2^, and $B_{3}$ = 1.03560653 $\times \;10^{2}$ μm^2^. Similarly, the dispersion relation for the TiO_2_ layer is given by [36]

(7) $$n_{{\mathrm{TiO}}_{2}}^{2}\left(\lambda \right)=A+\frac{B\lambda^{2}}{\lambda^{2}-C^{2}},$$

where constants *A*, *B*, and *C* have values of A=2.7655, B=2.2, and C=0.26524 μm.

The dispersion of the silver layer can be described by the complex dielectric function given by the Drude–Lorentz model [78]:

(8) $$\varepsilon_{\mathrm{Ag}}\left(\lambda \right)=\varepsilon_{\infty }-\frac{1}{\lambda_{p}^{2}\left(1/\lambda^{2}+i/\gamma_{p}\lambda \right)}-\sum_{j=1}^{5}\frac{A_{j}}{\lambda_{j}^{2}\left(1/\lambda^{2}-1/\lambda_{j}^{2}\right)+i\lambda_{j}^{2}/\gamma_{j}\lambda },$$

where the parameters are listed in Table 1.

A gaseous analyte is represented by humid air of a given relative humidity value, denoted here as RH, and its RI in the NIR spectra range is nearly non-dispersive [80] and can be approximated by a relation [13]:

(9) $$n_{\mathrm{Air}}=1+\left(272.7+1.024\;RH\right)\times 10^{-6},$$

**Figure 1:**
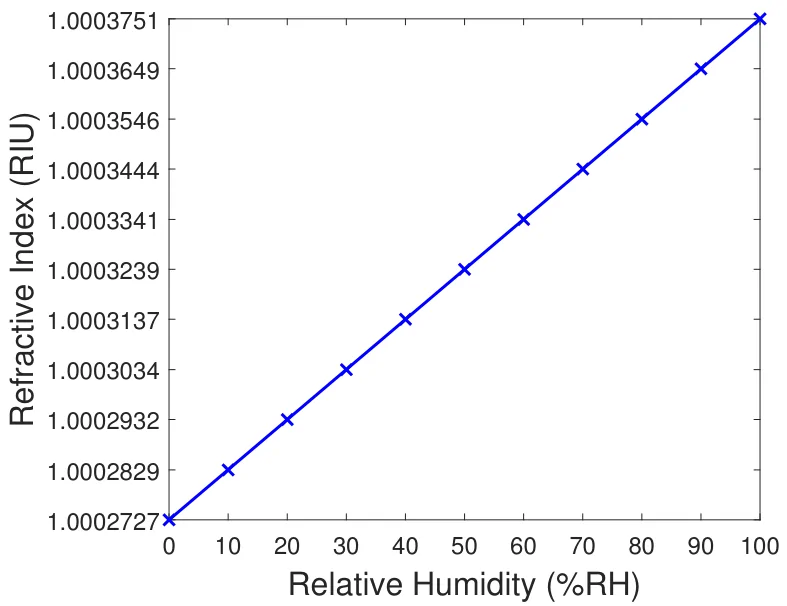
Refractive index of humid air as a function of its relative humidity.

This is shown in Figure 1, where a pressure of 1013 hPa and a temperature of 294 K are considered.

## 3. Proposed Measurement Scheme

**Figure 2:**
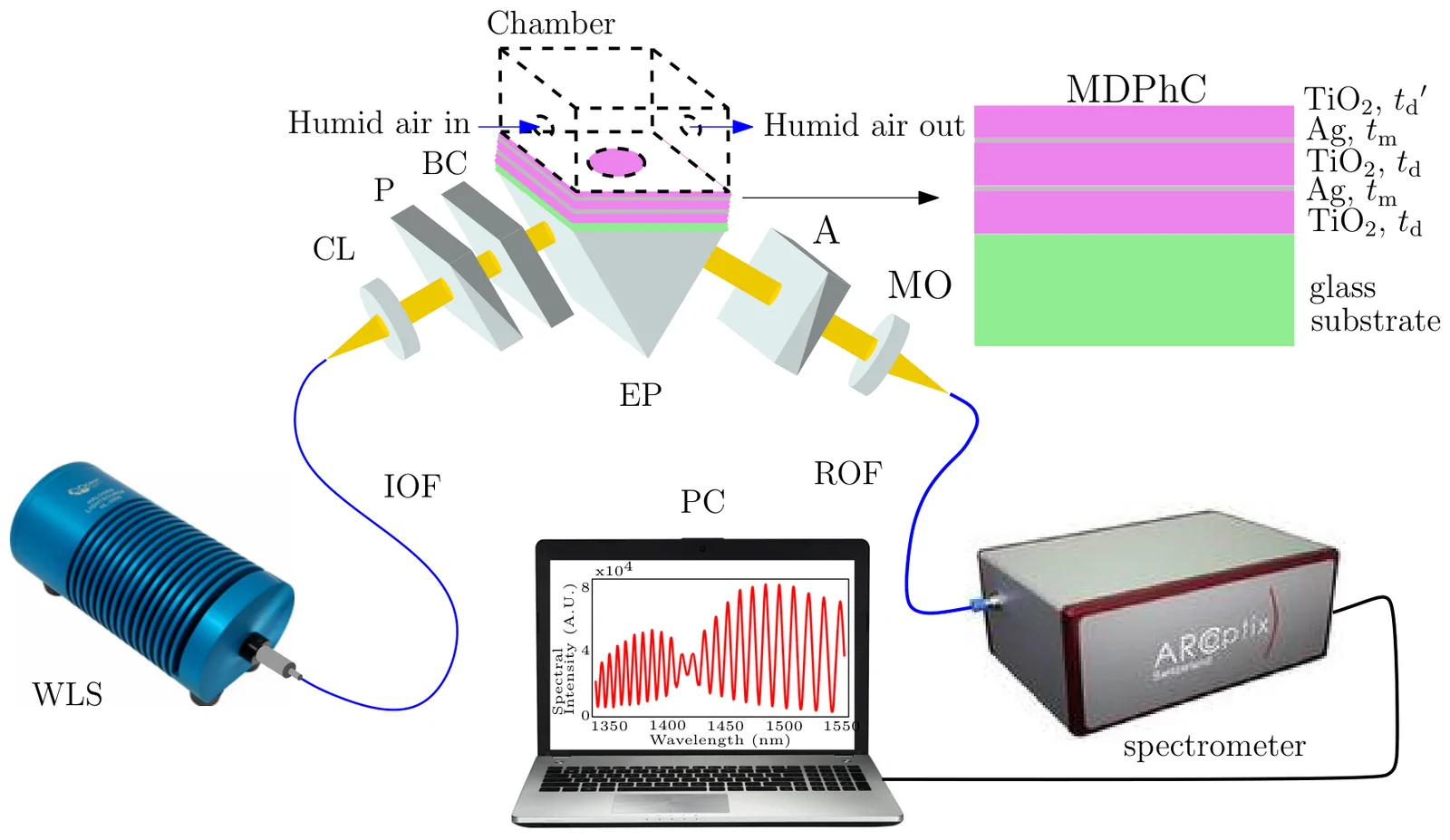
Schematic drawing of a proposed measurement scheme employing an MDPhC; white light source (WLS), input optical fiber (IOF), collimating lens (CL), polarizer (P), birefringent crystal (BC), equilateral prism (EP), analyzer (A), microscope objective (MO), read optical fiber (ROF), and personal computer (PC).

An experimental method that can be used for measuring the wavelength dependence of the phase difference $\Delta \left(\lambda \right)=\phi_{p}\left(\lambda \right)-\phi_{s}\left(\lambda \right)$ between *p*- and *s*-polarized components of an incident light beam subjected to the BLSW phenomenon is based on the spectral interference [81,82,83,84,85]. It is applied at a fixed angle of incidence and utilizes the proposed measurement scheme shown in Figure 2. In the same set-up, the spectral reflectances $R_{s,p}\left(\lambda \right)$ can be measured when birefringent crystal BC is removed. A collimated beam from a white light source (WLS) passes through the input optical fiber (IOF), collimating lens (CL), and polarizer (P) oriented at $45^{{}^{\circ}}$ with respect to the plane of incidence such that both *p*- and *s*-polarized components are generated. Next, the beam passes through a birefringent crystal (BC) whose optical axis is perpendicular to the plane of incidence, and the phase delays of the *p*- and *s*-polarized components are introduced. Finally, these components undergo, owing to reflection from a sensing structure consisting of a coupling equilateral prism (EP) and an MDPhC, the amplitude and phase changes that are related to the complex reflection coefficients given by Equation (3). The two orthogonally polarized components are mixed with the analyzer (A) and launched by the microscope objective (MO) into the read fiber of a spectrometer. The interference of the polarized components is resolved as a channeled spectrum in which the spectral phase difference Δ(λ) is inscribed, and it can be retrieved from the recorded channeled spectrum by using, for example, a windowed Fourier transform [83,86]. Alternatively, a commercial spectral ellipsometer, such as the J. A. Woollam RC-2 [87], can be employed to measure the ellipsometric phase Δ(λ).

## 4. Theoretical Results and Discussion

We performed a detailed analysis of the spectral reflectance and phase responses of an MDPhC for different angles of incidence and different wavelengths when the surrounding medium (analyte) was dry air with an RI of 1.0002727 RIU and when two bilayers of TiO_2_/Ag with a termination layer of TiO_2_ were considered. We took into account the dispersion of the prism and materials of the MDPhC given by Equations (6)–(8), while also assuming that the extinction coefficient for TiO_2_ had a value of 1.6 $\times \;10^{-3}$, as specified in previous papers [36,62]. Using a detailed analysis similar to our previous works [74,75], we demonstrated that the most pronounced dips in the reflectance spectrum were obtained for an Ag thickness $t_{m}=14$ nm, TiO_2_ thickness $t_{d}=410$ nm, and termination layer thickness ${t_{d}}^{\prime }=380$ nm.

**Figure 3:**
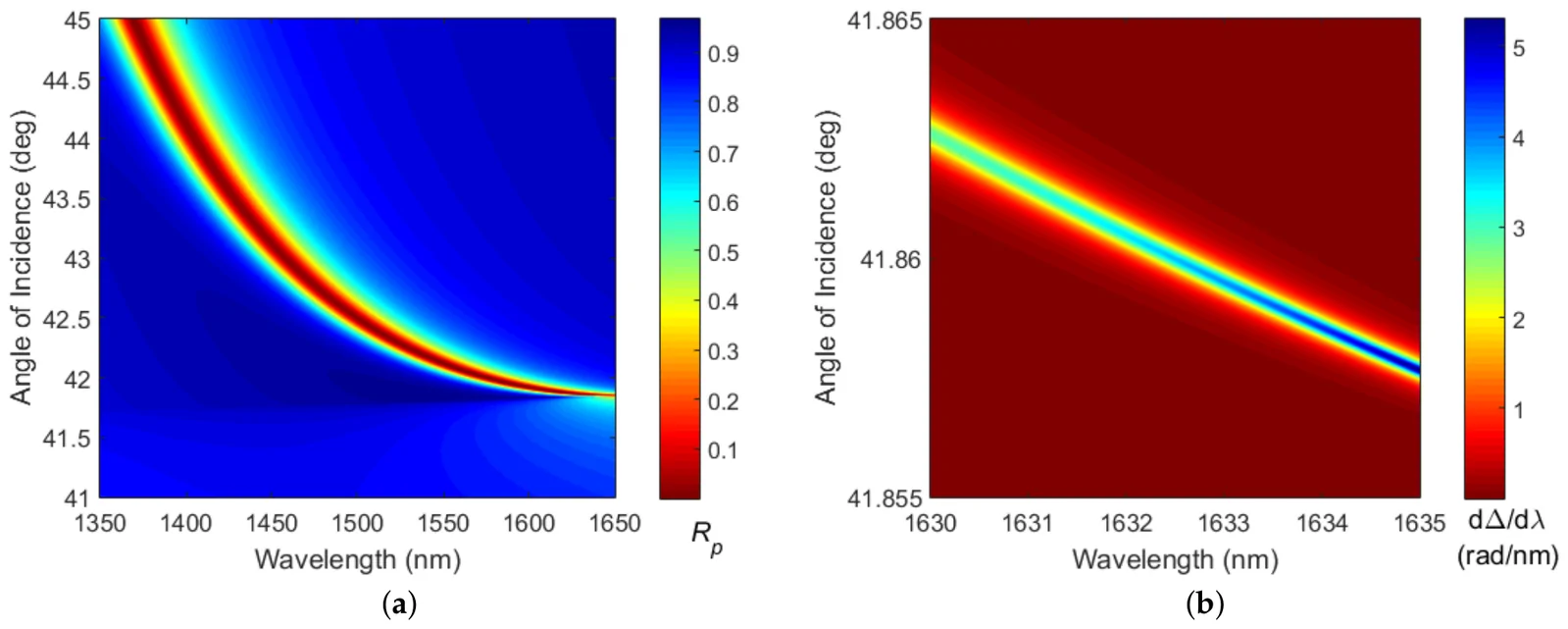
Calculated contour plots of the reflectance $R_{p}\left(\lambda \right)$ (**a**) and the derivative dΔ(λ)/dλ of the ellipsometric phase Δ(λ) (**b**) as a function of the angle of incidence and wavelength when the analyte RI is 1.0002727 RIU.

In order to have extensive insights on both the spectral reflectance and phase responses of the MDPhC with the sensitivity to the RI changes in the considered range being as high as possible, we first calculated contour plots of the reflectance for the TM wave with angles of incidence from $41^{{}^{\circ}}$ to $45^{{}^{\circ}}$ (degrees) and wavelengths in a range of 1350–1650 nm. From Figure 3a, it is clear that one mode up to the critical angle was present in the considered wavelength range, and it was associated with the BLSW [74,75]. The corresponding dip was of a nearly constant depth and width as the angle of incidence decreased.

Similarly, we calculated contour plots of the derivative dΔ(λ)/dλ of the ellipsometric phase Δ(λ) for the angles of incidence in a range of 41.855–$41.865^{{}^{\circ}}$ and wavelengths in a range of 1630–1635 nm, with the sensitivity to the RI changes in the considered range being as high as possible. Figure 3b shows that the derivative dΔ(λ)/dλ was increasing; in other words, the phase function was steeper as the angle of incidence decreased. This behavior is closely related to the spectral reflectance approaching a zero value.

**Figure 4:**
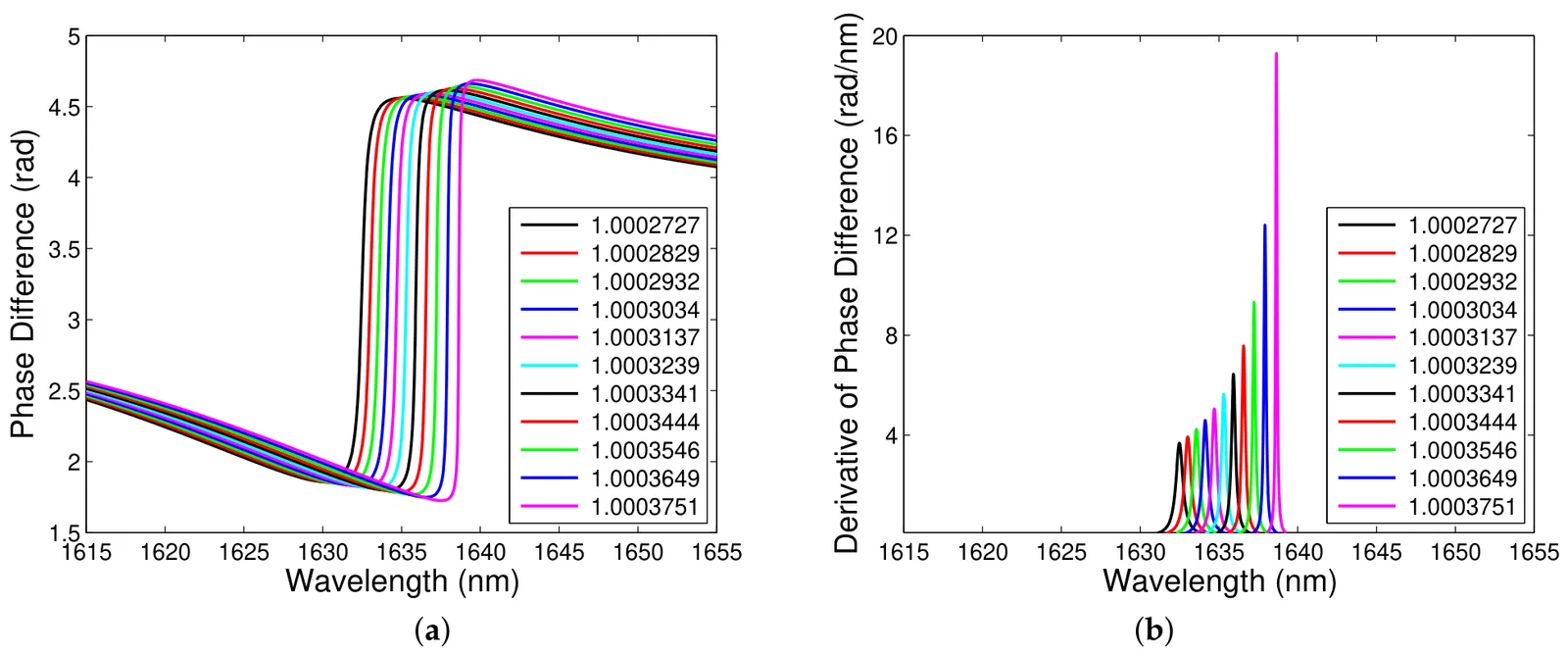
Theoretical spectral phase difference between TM and TE waves for different RIs of humid air (**a**) and the corresponding spectral derivatives (**b**). The angle of incidence is $\theta =41.86^{{}^{\circ}}$.

Based on the calculated contour plots of the derivative dΔ(λ)/dλ of the ellipsometric phase Δ(λ) as a function of the angle of incidence and wavelength, we chose the angle of incidence $\theta =41.86^{{}^{\circ}}$, and Figure 4a shows the wavelength dependence of the theoretical phase difference Δ(λ) for eleven samples of humid air when the RI changed in a range of 1.0002727–1.0003751 RIU or, equivalently, when the RH of the air changed in a range of 0–100 %RH with a step of 10 %RH. Figure 4a demonstrates that the BLSW excitation was manifested in the phase jumps that were steeper and with a greater amplitude as the RI of humid air increased.

The spectral derivatives of the theoretical phase differences as a function of the wavelength are depicted in Figure 4b for different RIs. The corresponding peaks are narrower, and their maxima reached higher values as the resonance wavelengths shifted toward longer wavelengths and as the RI of the humid air increased. The higher maxima are owing to the greater slopes of the spectral phase functions. The width of the spectral derivative of the phase difference for the last RI was nearly five times smaller than that for the first RI.

**Figure 5:**
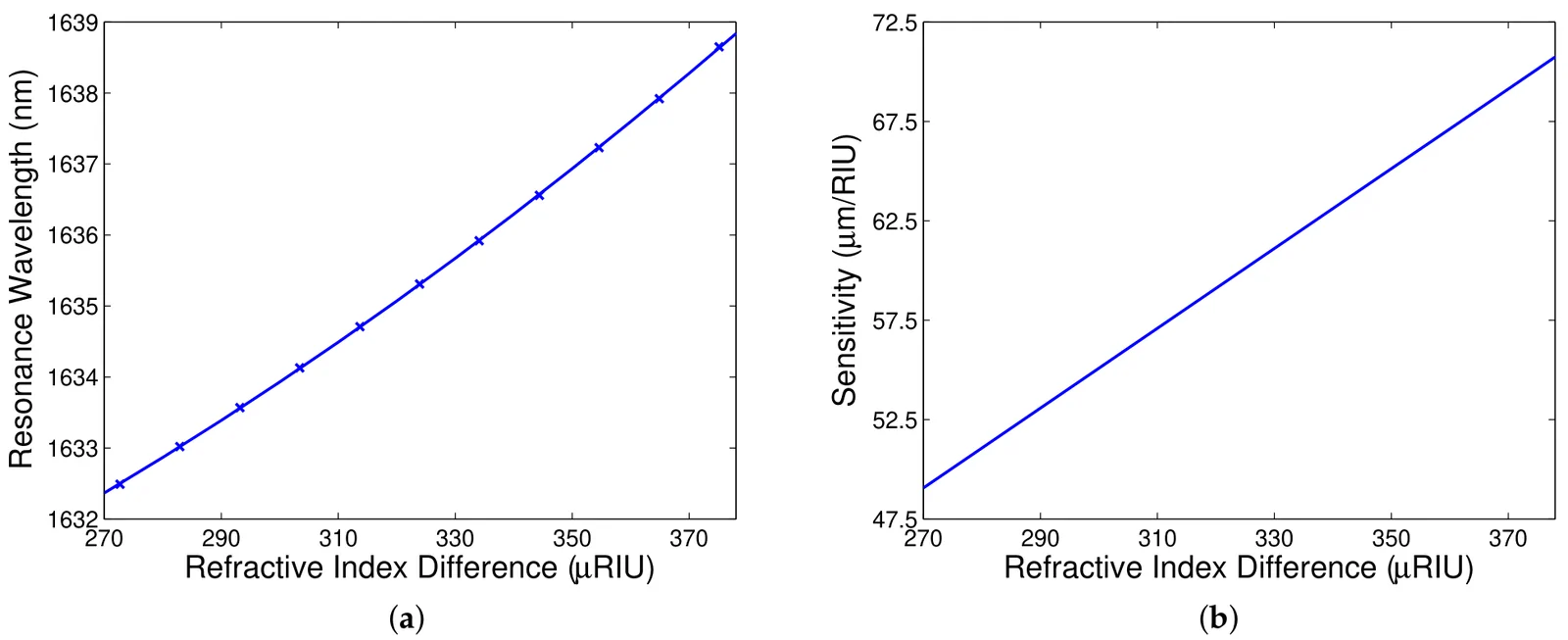
Resonance wavelength as a function of the RI difference in humid air, with a solid line as a fit (**a**) and the corresponding sensitivity function (**b**). The angle of incidence is $\theta =41.86^{{}^{\circ}}$.

The wavelength of the peak, the resonance wavelength, shifted toward longer wavelengths with increasing RI values for the humid air. The resonance wavelength $\lambda_{r}$, shown as a function of the RI difference n−1 in Figure 5a, where $n=n_{\mathrm{Air}}$, is depicted together with the fitting function (a second-order polynomial as the best fit). Such a nonlinear response means that the sensitivity to the refractive index $S_{n}$ was linearly RI-dependent, as shown in Figure 5b, because it is defined as

(10) $$S_{n}=\frac{\delta \lambda_{r}}{\delta n},$$

where $\delta \lambda_{r}$ is the change in the position of the peak with respect to the change in the analyte RI δn. The sensitivity $S_{n}$ was approximately in a range of 49–70 μm/RIU, and thus it was much higher than that of a BSW-based sensor (up to 3272 nm/RIU [88]) or other types of gas sensors [89]. To justify the sensitivity enhancement with the RI or RH of the air, we analyzed the fields of the Bloch-like SWs in TM polarizations and confirmed, in accordance with a previous analysis [74], that this was due to the increase in the penetration depth of the wave in the analyte. In addition, it should be stressed that when a greater angle of incidence was adjusted, a linear dependence of the resonance wavelength on the RI, advantageous from practical point of view, could be reached, but this was accompanied by the sensitivity decrease.

A quantity similar to the RI sensitivity $S_{n}$, namely the RH sensitivity $S_{\mathrm{RH}}$, can be defined analogously by replacing the RI *n* in Equation (10) with the RH. Consequently, for the above-mentioned values, the sensitivity $S_{\mathrm{RH}}$ was in a range of 0.050–0.072 nm/%RH.

The sensor performance was also evaluated in terms of LOD and FOM. The LOD is defined as the smallest RI that can be reliably detected, and it can be expressed by means of the sensitivity $S_{n}$ and the smallest spectral shift $\delta \lambda_{\min}$, which can be accurately measured as follows [90]:

(11) $$\mathrm{LOD}=\frac{\delta \lambda_{\min}}{S_{n}}.$$

Based on the spectral resolution $\delta \lambda_{\min}$ = 0.01 nm of a spectrometer [88], the LOD reached 1.4 × 10^−7^ RIU. Similarly, the LOD in the RH domain was expressed by replacing the RI *n* in Equation (11) with the RH, and it reached 0.14 %RH.

The FOM is defined as the sensitivity $S_{n}$ divided by the full-width half maximum (FWHM) of the resonance peak:

(12) $$\mathrm{FOM}=\frac{S_{n}}{\mathrm{FWHM}}.$$

The FWHM of the resonance peaks, shown in Figure 4b, varied approximately from 0.10 nm to 0.54 nm, and the FOM was in a range of (90,700–700,000) RIU^−1^. The FOM in the RH domain is expressed analogously, and it was in a range of 0.093–0.717 %RH^−1^. The sensors with these FOMs significantly outperformed available optical gas and RH sensors (see the next section).

The theoretical spectral phase difference Δ(λ), shown in Figure 4a, was characterized by an abrupt and incredibly steep change, resulting in a narrow and maximal spectral derivative dΔ(λ)/dλ or, equivalently, in a narrow and maximal resonance peak. The spectral width and magnitude of the resonance peak depend on the phase change near the resonance, and they are related to resonances approaching zero reflectance [57]. These specific conditions yielding to sharp phase changes near the resonance can be employed to radically improve the FOM of an optical sensor utilizing phase interrogation.

**Figure 6:**
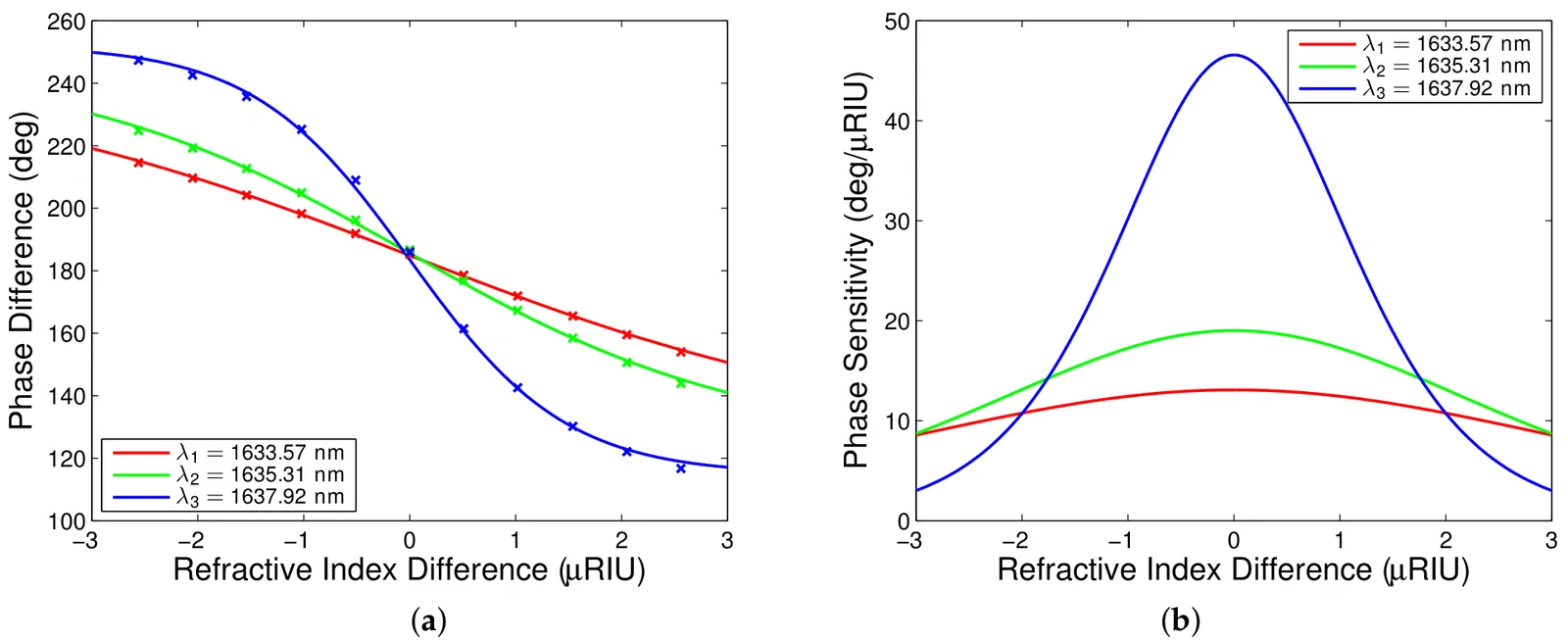
Phase difference $\Delta (\lambda_{0})$ as a function of the RI difference for humid air for three different wavelengths $\lambda_{0}$ with fits (**a**) and the corresponding sensitivity functions (**b**). The angle of incidence is $\theta =41.86^{{}^{\circ}}$.

To manifest the new approach, the phase difference $\Delta (\lambda_{0})$ was evaluated at a specific wavelength $\lambda_{0}$, and Figure 6a shows the quantity for three different wavelengths $\lambda_{0}$—1633.57, 1635.31, and 1637.92 nm, respectively—that corresponded to the resonance wavelengths for RHs of 20, 50, and 90 %RH, respectively. It can be clearly seen from Figure 6a, showing the phase difference $\Delta (\lambda_{0})$ as a function of the RI difference n−1. These curves are less proportional to the RI difference and become steeper as relative humidity increased or, equivalently, as the resonance wavelength $\lambda_{0}$ was redshifted.

The central parts of these curves can be approximated by linear functions and the phase sensitivity, defined by

(13) $$S_{\Delta }\left(\lambda_{0}\right)=\left|\frac{\delta \Delta (\lambda_{0})}{\delta n(\lambda_{0})}\right|,$$

reaching values of 12.2, 16.6, and 46.5 deg/μRIU at wavelengths of 1633.57, 1635.31, and 1637.92 nm, respectively. However, these sensitivities were accompanied by some limitations. The phase difference $\Delta (\lambda_{0})$ remained approximately linear only within a narrow RI range, and according to Figure 6a, this resulted in the RI measurement range (dynamic range) decreasing as the sensitivity increased.

To fit the theoretical phase difference $\Delta (\lambda_{0})$ as a function of the RI difference n−1, a polynomial fit was used, albeit with unsatisfactory results, and a better choice was an odd symmetric function, such as

(14) $$\Delta \left(\lambda_{0}\right)=a\tanh\left(bx\right)+c,$$

where *x* stands for n−1 and *a*, *b*, and *c* are fitting coefficients.

It is evident from Figure 6a that the fitting functions, whose parameters are listed in Table 2, agree well with the computed values, and they enabled evaluating the phase sensitivity $S_{\Delta }\left(\lambda_{0}\right)$ according to Equation (13) with

(15) $$S_{\Delta }\left(\lambda_{0}\right)=\left|ab[1-\tanh^{2}\left(bx\right)]\right|,$$

as depicted in Figure 6b. The phase sensitivity reached extremal values of 13.1, 19.0, and 46.6 deg/μRIU at wavelengths of 1633.57, 1635.31 and 1637.92 nm, respectively, and in the RH domain, the RH phase sensitivities $S_{\Delta }^{\mathrm{RH}}\left(\lambda_{0}\right)$ were 13.4, 19.5, and 47.7 deg/%RH, respectively.

Similar to the previous parts, the LOD at the wavelength $\lambda_{0}$ can be expressed by means of the sensitivity $S_{\Delta }\left(\lambda_{0}\right)$ and the smallest phase difference $\delta \Delta (\lambda_{0})$, which can be accurately measured as follows:

(16) $$\mathrm{LOD}\left(\lambda_{0}\right)=\frac{\delta \Delta (\lambda_{0})}{S_{\Delta }\left(\lambda_{0}\right)}.$$

If we use a rough estimate of the phase difference error $\delta \Delta (\lambda_{0})=$ 0.01°, which slightly exceeds that in [91] or is one order of the magnitude greater than previous estimates [67,70,92], then the lowest LODs at the corresponding wavelengths are $7.6\times 10^{-10}$, $5.2\times 10^{-10}$ and $2.1\times 10^{-10}$ RIU. In the RH domain, the lowest LODs were 7.5 $\times \;10^{-4}$, 5.1 $\times \;10^{-4}$, and 2.1 $\times \;10^{-4}$ %RH.

These values of the LOD, together with the sensitivities and FOMs specified above, significantly outperformed the parameters of the available optical gas and RH sensors (see Table 3 and Table 4). In addition, the results outperformed the liquid analyte sensors in terms of sensitivity and LOD: $4.63\times 10^{4}$ deg/RIU [57], $1.9\times 10^{6}$ deg/RIU and $2\times 10^{-8}$ RIU [60], $8.79\times 10^{4}$ deg/RIU and $9.28\times 10^{-8}$ RIU [67], $1.16\times 10^{4}$ deg/RIU and $8.62\times 10^{-7}$ RIU [68], $2.7\times 10^{4}$ deg/RIU and $1\times 10^{-7}$ RIU [70], and $3.4\times 10^{4}$ deg/RIU and $1.4\times 10^{-6}$ RIU [85].

To highlight the distinctive advantages of the performance of the proposed optical sensor based on the phase response of the MDPhC supporting BLSW, we compared the sensor with several recent optical gas and RH sensors from the point of view of the highest sensitivity and FOM, as well as the LOD (see Table 3 and Table 4). Along with the mentioned BSW- and TP-based sensors, these included sensors based on cavity modes (CMs) in 1DPhCs [34,93,94,95,96] or 2DPhCs [97], sensors utilizing lossy mode resonances (LMRs) [98,99], a PhC and plasmonic (PhC-P) hybrid topology [100], or a PhC micro-ring resonator (PhC-MRR) [101], as well as SPR effects [102,103].

**Table 3:** Comparison of recent optical gas sensors.

Method	RI Range (RIU)	$S_{n}$ (nm/RIU)	FOM (RIU^−1^)	LOD (RIU)	Year
CM (1DPhC)	1.0005087–1.0005387	21,688	–	–	2019 [94]
TP (PSi PhC)	1.00026–1.00046	190,000	360,000	$1.4\times 10^{-7}$	2020 [47]
BSW	1.00027–1.0070	3272	173.2	–	2020 [88]
PhC-P	1.000376–1.001132	1250	2083	–	2022 [100]
BSW	1.00029–1.00044	735.2	–	$1.14\times 10^{-4}$	2023 [31]
CM PTL	1.0001–1.0005	52,300	402,300	–	2023 [34]
PhC-MRR	1.00027300–1.00074873	6500	2960	–	2024 [101]
CM (2DPhC)	1.00037–1.00075	264	273	$3.66\times 10^{-4}$	2025 [97]
BLSW	1.0002–1.0022	28,000	1217	–	2026 [74]
BLSW	1.0002727–1.0003751	70,000	700,000	$1.4\times 10^{-7}$	This work
		**$S_{\Delta }\left(\lambda_{0}\right)$ (deg/RIU)**			
SPR	1–1.001	$1.7\times 10^{6}$	632.8	–	2019 [102]
SPR	1–1.001	$1.9\times 10^{6}$	632.8	–	2023 [103]
BSW	1.00029–1.00044	41.51	–	–	2023 [31]
BLSW	1.0002727–1.0003751	$4.66\times 10^{7}$	1637.92	$2.1\times 10^{-10}$	This work

**Table 4:** Comparison of recent optical RH sensors.

Method	RH Range (%RH)	$S_{\mathrm{RH}}$ (nm/%RH)	FOM (%RH^−1^)	LOD (%RH)	Year
1DPhC CM	11–84	0.296	–	–	2017 [93]
LMR	65–90	0.212	–	–	2019 [98]
LMR	30–90	0.636	–	–	2020 [99]
BSW	28.4–77.8	0.065	0.0013	0.15	2020 [88]
BSW	37–80	0.094	0.0086	–	2021 [33]
CM PTL	29.7–83.2	0.156	0.047	–	2023 [95]
TP PTL	34.0–80.0	0.090	0.004	–	2023 [43]
CM PTL	52.0–72.0	1.367	0.149	–	2023 [34]
CM PTL	31.2–56.2	0.444	0.178	0.022	2025 [96]
BLSW	0–100	0.072	0.717	0.14	This work
		**$S_{\Delta }^{\mathrm{RH}}\left(\lambda_{0}\right)$ (deg/%RH)**	**$\lambda_{0}$ (nm)**		
BSW	37–80	1.66	532	–	2021 [33]
BLSW	0–100	47.72	1637.92	$2.1\times 10^{-4}$	This work

It should be stressed that our theoretical calculations represent idealized performance, and manufactured MDPhCs will be inevitably affected by fabrication tolerances, the surface roughness, and material loss variations. Environmental noise and measurement errors can also substantially degrade sensitivity, widen resonance peaks, decrease the FOM and increase the LOD. These non-ideal effects will be discussed in detail under practical conditions. After a successful fabrication of the MDPhC, a more realistic estimation of an achievable sensitivity, FOM, and LOD could be provided.

## 5. Conclusions

In this theoretical paper, we presented a sensing concept based on the phase interrogation for BLSWs supported by an MDPhC in the Kretschmann configuration when the characteristics for sensing in humid air are substantially improved. This was illustrated by the sensitivities to the RI and RH of air and the FOMs as well, reaching values of 70 μm/RIU and 0.072 nm/%RH as well as 700,000 RIU^−1^ and 0.717 %RH^−1^, respectively. In addition, the LODs were substantially improved to values of 1.4 × 10^−7^ RIU and 0.14 %RH, respectively.

The analysis was extended to evaluate the phase sensitivities to the RI and RH of air at specific wavelengths, and at a wavelength of 1637.92 nm, they had values of 4.7 × 10^7^ deg/RIU and 47.7 deg/%RH, respectively, and the corresponding LODs were as low as 2.1 × 10^−10^ RIU and 2.1 × 10^−4^ %RH, respectively.

This sensing concept, based on the BLSW resonances in the phase domain of MDPhCs, demonstrated outstanding sensing characteristics. The advantageous concept, together with mechanically and chemically robust MDPhCs, is preferred in a simple, ultra-high-performance humidity sensing. Finally, the sensing concept is also offering the possibility of operation in aggressive environments and applicability to special gaseous analytes.

## Figures and Tables

**Table 1:** Parameters of the Drude–Lorentz dispersion formula for silver [79].

	Drude Term		Oscillators			
$\epsilon_{\infty }$	$\lambda_{p}$ (nm)	$\gamma_{p}$ (nm)	*j*	$A_{j}$	$\lambda_{j}$ (nm)	$\gamma_{j}$ (nm)
1	149.6970	25,830.0402	1	7.9247	1519.4141	319.0535
			2	0.5013	276.6886	2743.0131
			3	0.0133	151.4773	19,074.4912
			4	0.8266	136.5013	1353.5392
			5	1.1133	61.1060	512.5431

**Table 2:** Coefficients *a*, *b*, and *c* used for fitting the theoretical phase difference $\Delta (\lambda_{0})$.

$\lambda_{0}$ (nm)	*a* (deg)	*b* (μRIU^−1^)	*c* (deg)
1633.57	−58.23	0.2247	184.9
1635.31	60.56	−0.3140	185.6
1637.92	68.69	−0.6779	183.5

## Data Availability

Data are available from the corresponding authors upon reasonable request.
